# Spatiotemporal Changes of Ecosystem Service Value Determined by National Land Space Pattern Change: A Case Study of Fengdu County in The Three Gorges Reservoir Area, China

**DOI:** 10.3390/ijerph18095007

**Published:** 2021-05-09

**Authors:** Haozhe Zhang, Qingyuan Yang, Zhongxun Zhang, Dan Lu, Huiming Zhang

**Affiliations:** 1 School of Geographical Sciences, Southwest University, Chongqing 400045, China; ssaijj@email.swu.edu.cn (H.Z.); zhongxun2021@swu.edu.cn (Z.Z.); ludswu@email.swu.edu.cn (D.L.); zhm19981900@email.swu.edu.cn (H.Z.); 2 Chongqing Jinfo Mountain Kaster Ecosystem National Observation and Research Station, Chongqing 400715, China; 3 The State Cultivation Base of Eco-Agriculture for Southwest Mountainous Land, Southwest University, Chongqing 400045, China

**Keywords:** ecosystem services value, national land space pattern, spatiotemporal changes, Fengdu County, the Three Gorges Reservoir Area

## Abstract

Exploring the spatiotemporal change characteristics of ecosystem service value (ESV) under the influence of national land space pattern (NLSP) changes is of great significance for promoting the rational use of land resources and the optimization of ecosystems. In this study, Fengdu County in the Three Gorges Reservoir Area was selected as a case study. We analyzed the changes in NLSP using land use data from 1990, 2000, 2010 and 2018. Then, we used the equivalent factor method and exploratory spatial data analysis method to explore the spatiotemporal change characteristics of the ESV of Fengdu County. The results show that: (1) From 1990 to 2018, the changes in NLSP in Fengdu County generally manifested in the transformation of agricultural space into urban space and ecological space; (2) The spatiotemporal change of ESV is a process that positively responds to the increase in ecological space and negatively responds to the expansion of urban space. From 1990 to 2018, the total ESV of Fengdu County showed a trend of continuous growth, with a total increase of CNY 11.10 × 10^8^, and the change rate was 9.33%. The ESV gain area is mainly located along the Yangtze River and the southernmost part of the county, and the loss area is mainly located near the south bank of the Yangtze River; (3) ESV and its changes in Fengdu County have a significant positive spatial autocorrelation. The cold and hot spots of ESV change are mainly distributed along the Yangtze River and to the south of the Yangtze River. Therefore, it is suggested to integrate ESV as an important indicator into the decision-making of national land space planning. At the same time, it is necessary to strengthen the intensive use of urban space and protect the important ecological space from decreasing. Our study results provide useful insights for the development of regional NLS management and environmental protection policies. However, it is worth noting that the results of this paper are more applicable to areas where the terrain is dominated by mountains.

## 1. Introduction

Ecosystem services are the sum of life-sustaining products and services that humans obtain from ecosystems, which are closely related to human well-being and sustainable development [1]. The ecosystem supports and maintains the balance of the human living environment by regulating the climate and maintaining biodiversity [2]. It also provides the food and raw materials needed in life and production and brings entertainment and aesthetic enjoyment to human beings [3]. Ecosystem service value (ESV) is an important indicator to measure ecosystem service functions [4]. National land space (NLS) is the territorial space under the jurisdiction of national sovereignty and sovereign rights [5]. According to the categories of products provided, the NLS can be divided into urban space, with the main function of providing industrial products and service products, agricultural space, with the main function of providing agricultural products, and ecological space, with the main function of providing ecological products [6]. NLS includes resources and industrial elements such as land, labor, and minerals. Land is the supporting carrier of NLS, and the type, scale, and intensity of land use determine the national land space pattern (NLSP). At the same time, land is also a material provider of ecosystem services. Land use change is considered to be one of the main driving forces of changes in ecosystem services at regional and global levels because it reflects the coupling relationship between natural systems and human systems and profoundly affects the structure, function, and process of ecosystems [7]. Therefore, the NLSP change is an important factor leading to changes in ESV.

At present, with the increasing economic downturn and structural adjustment pressure, China is in a period of economic and social transformation. They face the challenges of tightening resource constraints, insufficient ecological environment carrying capacity, and incomplete institutional systems. It has become a top priority to coordinate the scientific protection and rational use of natural resources and to improve regional development quality. In this context, the Chinese government has changed the value orientation and measures of land resource allocation. The main functional area plan, land-use plan, and urban and rural plan are integrated into a unified national land spatial plan, and “multiple planning integration” is realized [8]. The emphasis of the national land spatial plan is to optimize the structure and layout of urban space, agricultural space, and ecological space. At the same time, the Chinese government has also proposed building a spatial planning system based on the core value orientation of pushing the development of an ecological civilization and practicing the “Two Mountains” Theory (Appendix A). Ecosystem services are related to environmental quality and human well-being and can be used as carriers for spatial plans to shape the value of natural resources [9,10]. ESV change can reflect the spatiotemporal effects and causality of planning decisions, which is conducive to deepening the understanding of the priority of space uses under the influence of multiple factors [11,12]. Therefore, the ESV is an important basis for promoting NLSP to conform to the concept of ecological civilization. Exploring the spatiotemporal change characteristics of ESV under the influence of NLSP change can provide a decision-making basis for national land spatial planning in the new era.

ESV is a hot topic of study because of the quick degradation of ecosystem services under intensive human disturbance [13]. Thus far, scholars have conducted significant work, primarily regarding the definition [1,7,14,15,16], classification [1,7,14,16,17], and valuation [1,18,19,20,21,22] of ecosystem services. Scientifically assessing the ESV can provide an important reference for ecosystem management [23,24], biological conservation [25], ecosystem service trade-offs [26,27], and ecological restoration [28,29]. At present, three main approaches have been widely applied to assess ecosystem services, including equivalent factors, productivity, and biomass [30]. Among them, the result of the equivalent factor method is presented in the form of monetary quantity, which can reflect the willingness of humans to pay for ecological services, with strong operability and wider applicability [31]. In 1997, Costanza et al. [1] divided the global ecosystem service functions, estimated the service value of each ecosystem item by item, and proposed the principles and methods of ESV estimation. Subsequently, scholars have studied the evaluation methods proposed by Costanza et al. and have explored and improved the theoretical methods of valuing ecosystem services [18,19,20]. Based on the research of Costanza et al. [1], Xie et al. [21,22] conducted a questionnaire survey among 200 professionals with ecological backgrounds and developed and improved the equivalent coefficient of ESV per unit area according to the actual situation in China, which has been used to evaluate the service value of land ecosystems. This method has the advantages of simple use, low data demand, high comparability of results, and comprehensive evaluation. It has been widely used in the study of ESV spatiotemporal change characteristics of administrative regions [32,33,34,35], natural regions [36,37,38,39,40], and economic regions [41,42,43] at different scales in China.

At present, the studies of NLS mainly focus on the optimization and regulation of NLSP based on the relationship between land systems and the internal environment of natural systems [44,45]. The partitioning and adjustment of regional NLS are studied from the aspect of multifunctional land characteristics [10,46]. China’s territorial imbalance has intensified [47,48] and there has been a coexistence of insufficient space for the development and over-development of space; therefore, the optimization and control methods of NLSP based on regional functional suitability and resource and environmental carrying capacity have gradually become a hot topic [49,50].

In summary, we find that the existing research methods are gradually mature and the research results are constantly enriched, but still need to be improved in the following three aspects: (1) Most scholars believe that land use/cover change (LUCC) is the main reason for the change in ESV, mainly studying the spatiotemporal change characteristics of ESV based on the perspective of LUCC [32,33,34,35,37,38,41,51]. However, attention has rarely been paid to changes in the urban–agricultural–ecological functions of NLSs. No scholar has explored the spatiotemporal change characteristics of ESV based on the perspectives of ecology, agriculture, and urban spatial changes. Therefore, the relevant studies have limited guiding value for national land spatial planning; (2) The exploratory spatial statistical analysis method based on grid cells can effectively express the spatial change characteristics of ESV and can also more accurately describe the local impact of changes in NLSP on ESV, but it has only been applied by a few studies [52,53]; (3) In terms of case area selection, most of the current studies have selected provincial or city-level administrative areas, while there are few studies on county-level administrative areas. Although these studies are typical, their results are insufficient for the practical significance of county-level administrative units.

The Three Gorges Reservoir Area (TGRA) is an important ecological barrier in the Yangtze River Basin. Its ecological environment is not only directly related to the long-term safe operation of the Three Gorges Dam (TGD) and the stable enrichment of millions of immigrants, but is also related to the ecological security and sustainable development of the whole Yangtze River Basin. However, the terrain of the TGRA is dominated by mountains and hills, with serious problems such as a fragile ecological environment, nonpoint source pollution, landscape fragmentation and soil erosion, and the contradiction between humans and land is prominent [54]. Located in the hinterland of the TGRA, Fengdu County is faced with multiple problems, such as immigration relocation, hollowing out of the countryside, rapid urban expansion, and deterioration of the ecological environment. The rational development of NLSs and ecological environmental protection are facing huge challenges. In view of this, we selected Fengdu County as the study area, using land use data from 1990, 2000, 2010, and 2018 to analyze the NLSP change based on the perspectives of urban, agricultural, and ecological spaces. Then, we used the equivalent factor method and exploratory spatial data analysis method to explore the spatiotemporal change characteristics of ESV in Fengdu County. The specific objectives of this study are described as follows: (1) to characterize the change characteristics of NLSP in Fengdu County from 1990 to 2018; (2) to analyze the spatiotemporal change characteristics of ESV in Fengdu County; and (3) to explore the spatial autocorrelation characteristics of ESV and its change.

## 2. Materials and Methods

### 2.1. Study Area

**Figure 1:**
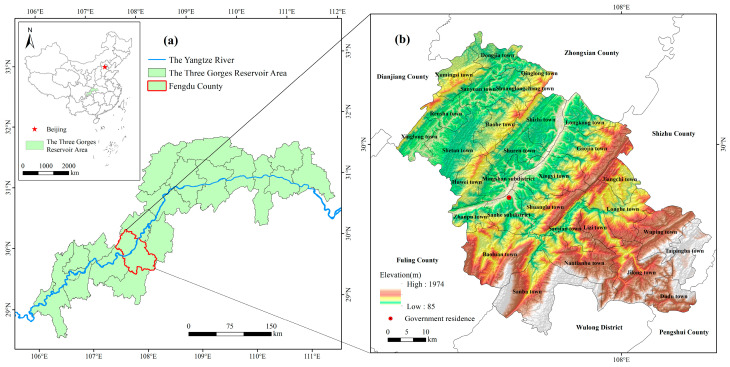
The study area: (**a**) the location of Fengdu County in the Three Gorges Reservoir Area; (**b**) the administrative divisions and elevation distribution of Fengdu County.

Fengdu County of Chongqing city is located in the upper reaches of the Yangtze River and in the hinterland of the TGRA. The geographical coordinates are 107°28′–108°12′ E and 29°33′–30°16′ N (Figure 1). The terrain is dominated by mountains, followed by hills and only a few flat dams in mountains and river valleys, showing a general pattern of high south and low north. The land uses are mainly cropland and forest, with abundant forest resources and diverse ecosystems. Fengdu County has a total area of 2900.86 km^2^, with 2 subdistricts, 23 towns, and 5 townships. In 2018, the per capita GDP of Fengdu County was CNY 40,400, the per capita disposable income was CNY 21,300, and the population urbanization rate was 46.48%. Since the 21st century, the immigrant population in Fengdu County has been larger than the non-immigrant population, and the number of total permanent population dropped from 671,100 in 2000 to 585,200 in 2018. The outflow population mainly comes from rural areas. Between 2000 and 2018, the number of rural population decreased by 210,600.

### 2.2. Data Sources

The data used in this paper mainly include: (1) Land use data obtained from the Chinese Academy of Sciences Resource and Environmental Science Data Center (https://www.resdc.cn/ (accessed on 1 September 2020)), including four periods of land use data in 1990, 2000, 2010 and 2018, with a spatial resolution of 30 m. The types of land use in the study area include 6 primary categories (cropland, forest, grassland, water, built-up and unused land) and 17 secondary categories (omitted here); (2) Net Primary Productivity (NPP) data were obtained from the National Aeronautics and Space Administration (https://www.nasa.gov/ (accessed on 1 September 2020)), and soil conservation simulation and precipitation data were obtained from the National Earth System Science Data Sharing Service Platform (https://www.geodata.cn/ (accessed on 1 September 2020)). These data were used to modify the ecosystem service value equivalent factors; (3) The data on the yield per unit area and average price of rice, wheat and corn are from the “China Agricultural Product Price Survey Yearbook”, which is used for the calculation of the ESV of a standard equivalent factor. The other socioeconomic data required for this article come from the “China Statistical Yearbook”, “Chongqing Statistical Yearbook”, and “Fengdu Yearbook” of the corresponding years.

### 2.3. Methods

#### 2.3.1. Quantitative Analysis of National Land Space Pattern Change

(1). National Land Space Classification

**Figure 2:**
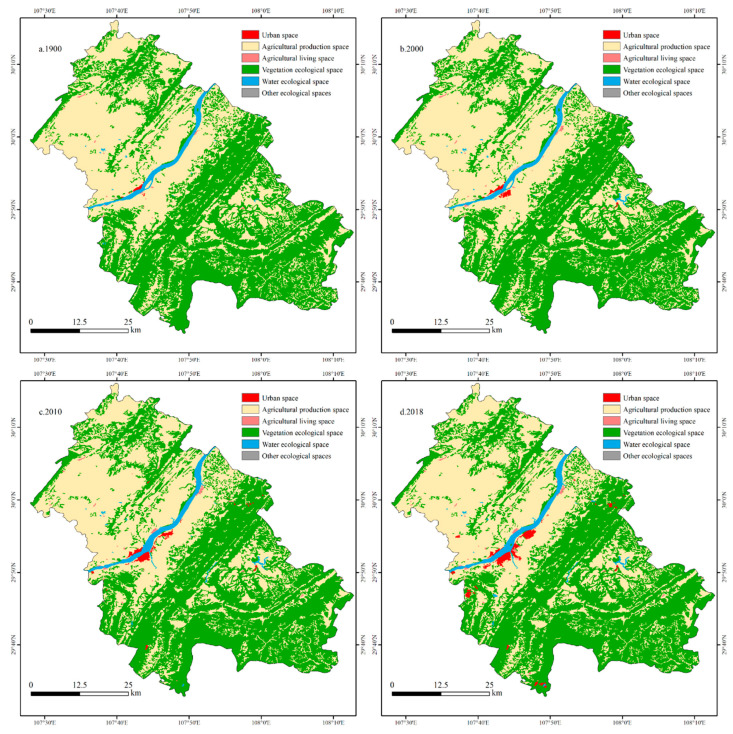
Spatial distribution of NLS in Fengdu County from 1990 to 2018.

NLS classification is the process of considering the various functions of land in the natural environment and the development of human society, and dividing it into several functional areas [55]. Among the multiple functions of NLS, ecological function is the premise and foundation of other functions [56]. Based on the “Main Functional Area Planning” issued by the State Council of China, we took the strengthening of the basic status of ecological functions as the goal and established the NLS classification system of Fengdu County [57]. The classification system included 3 primary categories and 6 secondary categories (Table 1, Figure 2). As the spaces with different functions in the urban space are closely connected and the boundaries are blurred, the urban space is not divided into secondary categories. It is worth noting that the Chinese government added Fengdu County as an important area for soil conservation in the “National Ecological Function Zoning of China” program, and made the protection and restoration of vegetation as the focus of the regional ecosystem management. Therefore, we classified forest and grassland as vegetation ecological space, which not only emphasizes its ecological attributes, but is also conducive to the management of local soil erosion and rocky desertification. In addition, we classified the bare land as other ecological spaces, to prevent the blind adjustment of these lands into cropland and built-up in the process of subsequent NLS development, which is conducive to promoting the natural ecological restoration of degraded land.

(2). National land Space Transition Matrix

The land-use transition matrix can not only reflect the initial and final land type structure of the study area, but also describes the detailed changes of land use in the study area, including the source, composition, and direction of the changes [58]. Based on the theory of the land use transition matrix, we established the NLS transition matrix to analyze the change structure and direction of NLS during the study period; its mathematical expression is:

(1) $$S_{ij}=\left|\begin{array}{cccc} S_{11} & S_{12} & \cdots & S_{1n}\\ S_{21} & S_{22} & \cdots & S_{2n}\\ \vdots & \vdots & \vdots & \vdots \\ S_{n1} & S_{n2} & \cdots & S_{nn} \end{array}\right|,$$

In the formula, i and j are the NLS types in the previous stage and later stage, n is the number of NLS types, and $S_{ij}$ is the area of the ith NLS type transferred to the jth NLS type during the research period. We used the raster calculator tool in ArcGIS 10.7 to calculate the NLS transition matrix, and to realize the visualization of the results. The formula is as follows:

(2) $$G=10G_{a}+G_{b},$$

In Equation (2), G is the new NLS unit code formed by the change in NLS during the study stage, $G_{a}$ is the NLS unit code in the previous stage, $G_{b}$ is the NLS unit code in the later stage, and the codes for US, APS, ALS, VES, WES, and OES are 1, 2, 3, 4, 5, and 6, respectively.

#### 2.3.2. Calculation of Ecosystem Services Value

This study used the equivalent factor method proposed by Xie et al. [59] to measure ESV. In this method, ecosystem services are divided into 4 primary categories and 11 secondary categories. Specifically, provisioning services include food supply, raw material supply, and water supply. Regulating services include air quality regulation, climate regulation, waste treatment, and regulation of water flows. Supporting services include erosion prevention, maintenance of soil fertility, and habitat services. Cultural services include cultural and amenity services. The equivalent value per unit area of food production of cropland was set to 1, and the equivalent value per unit area of other ecosystem services could be quantified by comparison with the standard value of 1. Generally, the economic value provided by natural ecosystems without human input is approximately 1/7 of the economic value of food provided by existing farmland per unit area. From 1990 to 2018, the annual average yield of the three main food crops (rice, corn, and soybean) in Fengdu County was 3589.90 kg/hm^2^, and the average price of the three main food crops in 2018 was 2.98 CNY/kg. Therefore, the unit value of the equivalent factor ($E_{n}$) was 1528.27 CNY/hm^2^, obtained using the following equation:

(3) $$E_{n}=1/7\;PQ,$$

In the formula, P is the annual average grain yield, and Q is the average grain price.

Considering the regional heterogeneity of the internal structure and external form of the ecosystem, there are obvious differences in ecosystem service functions and value in different regions [60]. The table of the ESV equivalent factors proposed by Xie et al. is applicable at the national scale. If applied directly to regional ESV research, major errors may occur. Therefore, we adjusted the ESV coefficients to suit the ecological characteristics of Fengdu County. The ecosystems were divided into 6 primary categories and 14 secondary categories in the study of Xie et al. [59]. Land use types cannot correspond to ecosystem types one by one; therefore, we selected the closest land use type for equivalent evaluation according to the actual situation of the study area. Specifically, the equivalent factors of the corresponding ecosystem types were used for paddy field, dry land, water, and bare land. Broadleaf forests are the main types of forest, and shrub grass is the main type of grassland in Fengdu County [61]; we used the equivalent factors of broadleaf forest and shrub grass as the representatives of forest and grassland, respectively. Simultaneously, we adjusted the ESV equivalent factors based on the spatial distribution raster data of national net primary productivity (NPP), precipitation per unit area, and soil conservation [22]. Among these raster data, the NPP, precipitation per unit area, and simulated soil conservation of each grid are the average values of 1990, 2000, 2010 and 2015, calculated by the raster calculator tool in ArcGIS 10.7. Thus, the ESV coefficients per unit area were calculated according to Equation (4).

(4) $$VC_{f}=\{\begin{array}{c} D_{f1}\times E_{n}\times \bar{B}/\bar{B_{t}}\;\mathrm{or}\\ D_{f2}\times E_{n}\times \bar{W}/\bar{W_{t}}\;\mathrm{or}\\ D_{f3}\times E_{n}\times \bar{E}/\bar{E_{t}}\;\;\; \end{array},$$

In the formula, $VC_{f}$ is the ESV coefficient per unit area of the fth ecological service type of a certain ecosystem in the study area, $D_{f1}$, $D_{f2},\;$and $D_{f3}$ are the equivalent factors of the ecosystem service function related to NPP, precipitation, and soil conservation, respectively [61], $\bar{B}$, $\bar{W}$, and $\bar{E}$ are the average NPP, precipitation per unit area, and simulated soil conservation in the study area, respectively, and$\;\bar{B_{t}}$, $\bar{W_{t}}$, and $\bar{E_{t}}$ are the national average NPP, precipitation per unit area, and soil conservation simulation quantity, respectively. In addition, several researchers believe that “built-up” does not belong to natural ecosystems and assign an ESV value of zero to built-up areas [62,63]. However, due to the strong disturbance of human activities, built-up spaces have a huge impact on regional ecosystem services. Human activities in built-up areas consume food and water, and emit exhaust gas, wastewater, and solid waste at the same time, which have a negative impact on provisioning services and regulating services. However, built-up zones have the function of maintaining soil, and also add the values of appreciation and entertainment; thus, it has a positive impact on supporting services and cultural services [51]. In this study, we determined the ESV coefficient per unit of built-up area based on the research of the Chengdu–Chongqing Economic Zone by Yuan et al. [64]. Finally, we obtained the ESV per unit area for Fengdu County (Table 2).

The formulae for estimating the ESV are as follows:

(5) $$ESV_{f}=\sum A_{k}\times VC_{fk},$$

(6) $$ESV=\sum A_{k}\times VC_{k},$$

where $ESV_{f}$ is the value of the fth ecological service type, ESV is the total value of ecosystem services in the study area, $A_{k}$ is the area of the kth land use type, $VC_{fk}$ is the ESV per unit area of the fth ecological service type of the kth land use type, and $VC_{k}$ is the ESV per unit area of the kth land use type.

Based on this, the formula for calculating the ESV of different NLS types is as follows:

(7) $$ESV_{g}=\sum S_{k}\times VC_{k},$$

In Equation (7), $ESV_{g}$ is the ESV of a certain type of NLS, and $S_{k}$ is the area of the kth land-use type included in this type of NLS.

#### 2.3.3. Exploratory Spatial Data Analysis

Exploratory spatial data analysis is a collection of techniques for describing and visualizing spatial distributions, determining atypical locations or spatial outliers, discovering spatial associations, clusters, or hot spots, and inferring spatial characteristics or other forms of space heterogeneity [65]. In this study, the global spatial autocorrelation analysis method and hot spot analysis method are adopted to explore the spatial autocorrelation characteristics of ESV. We calculated Moran’s *Ι* value, which is used to describe the global spatial autocorrelation characteristics of ESV. At the same time, we calculated Getis–Ord Gi* statistics to describe the spatial locations of “cold spots” and “hot spots” of ESV changes. These statistics are used to reveal the spatial clustering pattern of the high and low values of ESV change. The Spatial Autocorrelation (Moran’s *Ι*) and Hot Spot Analysis (Getis–Ord Gi*) tools in ArcGIS 10.7 software were used for analysis.

## 3. Results

### 3.1. National Land Space Pattern Change

#### 3.1.1. The Area Change of National Land Space

According to the land use data for the four periods of 1990, 2000, 2010 and 2018, the area change of NLS was calculated (Table 3), and the results showed that Fengdu County is dominated by ES, which accounted for approximately 53%, followed by AS, which accounted for more than 45%, while US is the smallest, accounting for less than 1%. From 1990 to 2018, the US of Fengdu County expanded significantly (1165.64%). There was a slight decrease in AS (−4.15%) and a small increase in ES (2.18%). From the changes in the secondary types, it can be seen that US, ALS, VES and WES have increased, while the rest of the space has decreased. Among them, the US has the fastest growth rate (41.69%/a), which reflects the intense urbanization process that Fengdu County has experienced in the past 28 years. The growth rate of ALS is 5.47%/a, which is second only to US. From the changes of different stages, it can be seen that US, ALS and WES continue to increase. US is gradually increasing, and the increase in ALS at each stage is relatively stable. The WES increased explosively from 2000 to 2010, mainly because the Three Gorges Reservoir began to store water at this stage, which led to the expansion of the water area of the Yangtze River and its tributaries. APS and OES continued to decrease, and both had the largest decrease from 2000 to 2010. The VES increased sharply from 2000 to 2010, which may be related to the effective implementation of policies such as the Grain for Green Project, the Natural Forest Protection Program, and the policy of rocky desertification control at this stage.

#### 3.1.2. National Land Space Transition

Table 4 shows the NLS transition matrix of Fengdu County. It can be seen that VES has the largest transfer-in area, which is mainly from APS (100.45 km^2^). Next is WES, which mainly comes from APS (8.35 km^2^) and VES (5.04 km^2^). US is mainly transferred from APS (18.24 km^2^) and VES (5.24 km^2^), and ALS is mainly transferred from APS (5.39 km^2^). We found that the hot spot of transfer in Fengdu County is the transfer of APS to VES and US.

**Figure 3:**
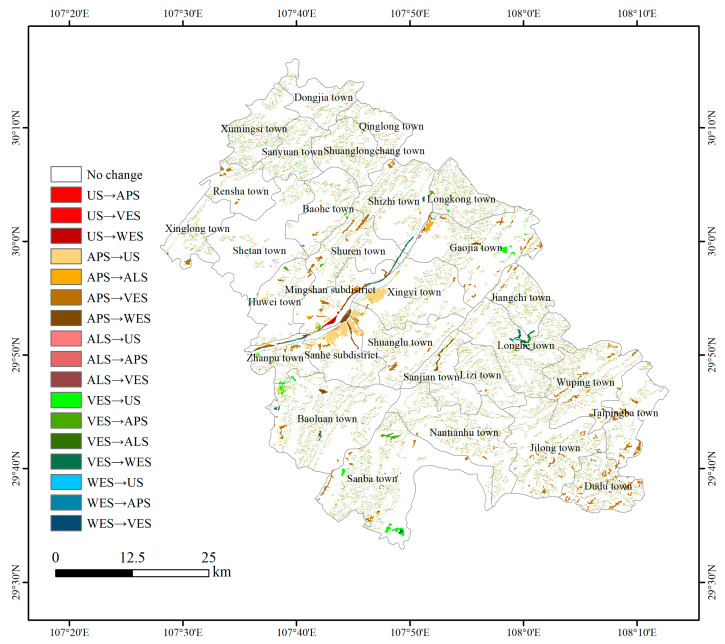
NLS transition map in Fengdu County from 1990 to 2018.

From the spatial distribution of NLS in Fengdu County (Figure 2), it can be seen that, initially, the APS was roughly distributed in mountain valleys and gentle slope areas. These areas have a flat terrain and good hydraulic conditions, which are suitable for agricultural production. VES were scattered in mountains and valleys. The complex natural environment in these areas restricts human activities and provides a good condition for the restoration of vegetation. The WES mainly includes the Yangtze River and Long River. The US and ALS are mainly distributed along the Yangtze River. According to the NLS transition map in Fengdu County from 1990 to 2018 (Figure 3), the most obvious NLS change was the expansion of US, which is mainly distributed in the north of the Sanhe subdistrict, northwest of Shuanglu town, and northwest of Xingyi town. This is mainly because Fengdu County started the construction of a new county town on the south bank of the Yangtze River after the old county town was flooded. Next, transitions between APS and VES occurred in Fengdu County at a large scale, but the locations were relatively scattered. Among them, the transfer from APS to VES in some areas with steep slopes in townships, such as Shuren Town, Dudu Town, Jilong Town, Wuping Town, and Taipingba Township is obvious. These areas were unsuitable for farming, therefore they were the key areas for the Grain for Green Project. The expansion of WES was distributed along the Yangtze River, mainly due to the operation of the TGD. The construction of many small reservoirs in Gaojia town, Longhe town and Baoluan town also resulted in the transfer of a small amount of APS into WES. The transfer-in of ALS was mainly distributed in northern Zhanpu town, eastern Mingshan town, and northeastern Gaojia town.

### 3.2. Spatiotemporal Changes of Ecosystem Service Value

#### 3.2.1. The Changes of Ecosystem Service Value

The ESVs of Fengdu County in 1990, 2000, 2010, and 2018 were calculated in combination with the ESV per unit area (Table 5). The results show that the total ESV of Fengdu County showed a trend of continuous growth, with a total increase of CNY 11.10 × 10^8^, and the change rate was 9.33%, during the 1990–2018 period. From the perspective of different stages, the total ESV growth rate of Fengdu County was the largest from 2000 to 2010, which was mainly due to the rapid expansion of the scale of VES and WES during this period. Among the four primary types of ecosystems services, the value of regulating services had the largest contribution to the total ESV. In the past 28 years, the value of the four ecosystem services has increased. Among them, cultural services (CNY 4.66 × 10^8^) have the most value growth, followed by provisioning services (CNY 3.97 × 10^8^) and regulating services (CNY 1.97 × 10^8^); supporting services (CNY 0.49 × 10^8^) have the least value growth.

The largest ESV per unit area is for the water and the lowest is for the unused land, whereas the ESV per unit area of the built-up is negative (Table 2). According to Equation (7), the ESV of different NLS types was calculated (Table 6), and it was found that the VES is the main contributor to the ESV of Fengdu County and has contributed more than 60% during the four periods; the next highest contributor is the WES, at approximately 25%. The contributions of the other NLS types, which have smaller areas, were relatively low; especially for OES, which was close to zero. From the perspective of ESV changes, we observed that the ESV of VES and WES increased by CNY 3.07 × 10^8^ and CNY 8.78 × 10^8^, respectively. The ESV of the remaining space types all declined. Among them, the ESV of APS decreased the most (CNY 0.57 × 10^8^), followed by US (CNY 0.16 × 10^8^) and ALS (CNY 0.03 × 10^8^). In summary, the ESV loss caused by the expansion of the construction space (US and ALS) and the reduction in APS is offset by the ESV gain caused by the increase in ES. The significant increase in the ESV of the WES is the main reason for the increase in the total ESV of Fengdu County.

#### 3.2.2. The Spatial Change of Ecosystem Service Value

**Figure 4:**
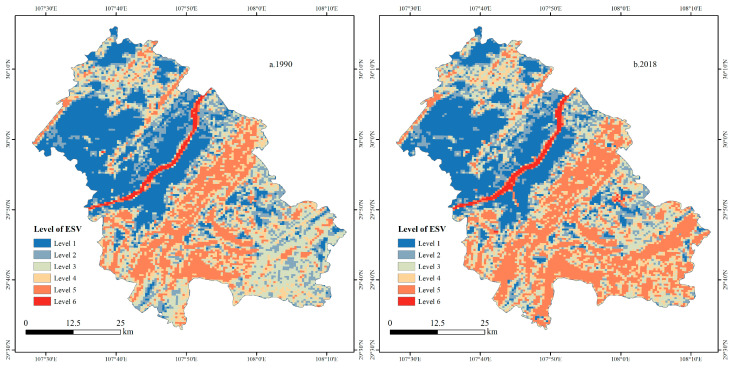
Spatial distribution of ESV in Fengdu County, 1990 and 2018.

To further analyze the spatial variation characteristics of ESV, the Fish net tool in ArcGIS 10.7 was used to divide the administrative area of Fengdu County into 12,052 square cells with a side length of 500 m. We calculated the ESV of each grid and used natural breaks to divide it into six levels from high to low. Among them, an ESV of level one was the lowest, and an ESV of level six was the highest (Figure 4). The results showed that the spatial difference in ESV in Fengdu County was obvious. Due to the vast water area, the Yangtze River is a concentrated area with the highest value of ecosystem services. In addition, the ESV in Fengdu County has obvious spatial distribution characteristics, with the Yangtze River as the boundary, high in the south and low in the north.

**Figure 5:**
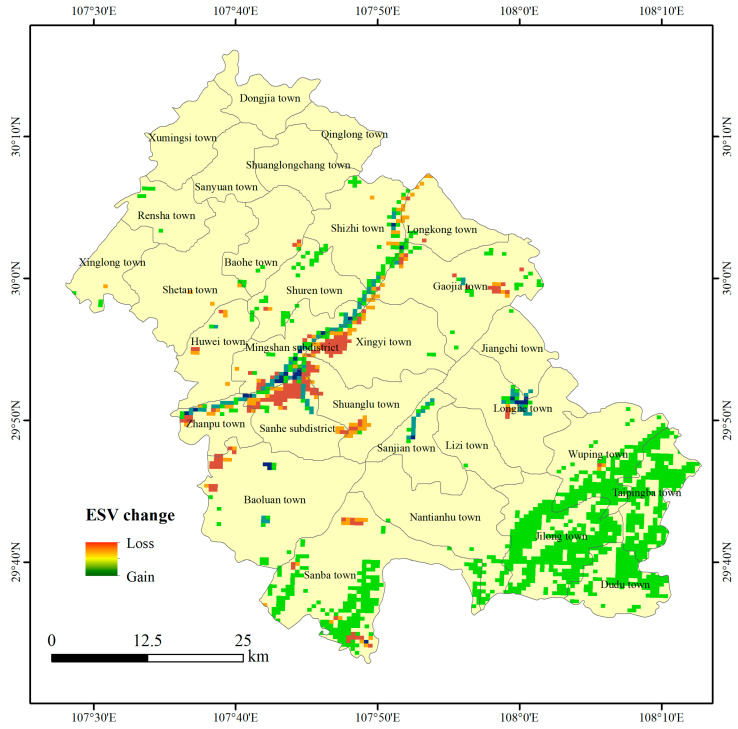
Spatial distribution of ESV change in Fengdu County from 1990 to 2018.

Figure 5 is the spatial distribution map of ESV changes in Fengdu County from 1990 to 2018. The changes of ESV in Fengdu County were mainly distributed along the Yangtze River and the southernmost part of the county, while the changes of ESV in other areas were not obvious. The ESV gain area along the Yangtze River was distributed in a zonal pattern, mainly located to the north of Zhanpu town and the south of Mingshan subdistrict, Shuren town and Shizhi town. The loss areas were distributed in blocks, mainly located in the northern Sanhe subdistrict, Shuanglu town, Xingzhi town and Gaojia town. Among them, the gain in ESV was mainly due to expansion of the water area of the Yangtze River, and the loss of ESV was mainly due to the continuous encroachment of other spaces by US and ALS. The areas with high ESV values in Sanba Township, Jilong Township, Dudu Township, Taipingba Township and Wuping Town in the southernmost part of Fengdu County increased, which was mainly due to the transition of a large amount of grassland in the ES into forest. There were also some scattered ESV gain and loss areas. Among them, the loss of ESV was mainly due to the transformation of APS and VES into US and ALS. The main reasons for the gain of ESV on both sides of the Yangtze River are different. In the north of the Yangtze River, it was mainly due to the transformation of APS into VES. However, in the south of the Yangtze River, it was mainly due to the transformation of VES into WES.

#### 3.2.3. Spatial Autocorrelation Characteristics of Ecosystem Service Value

(1). Global Spatial Autocorrelation Analysis

The spatial correlation characteristics of ESV are shown in Table 7. It can be seen that the global Moran’s *Ι* of ESV and ESV changes in each stage were all greater than 0 and significant at the threshold level of 1%, indicating that ESV and its changes in Fengdu County were not randomly distributed but positively correlated. This indicates that the spatial distribution of ESV and its changes showed strong spatial clustering characteristics. The Moran’s *Ι* of ESV first decreased from 0.6324 to 0.6214, and then continually rose to 0.6437. This indicates that the clustering characteristics of ESV distribution were weakened from 1990 to 2000, but the clustering characteristics of ESV distribution were enhanced from 2000 to 2018 under the influence of the construction of the Three Gorges Project, the implementation of macro policies, and the rapid development of urbanization; the Moran’s *Ι* of ESV changes at different stages showed “up–down” fluctuation characteristics. From 2000 to 2010, the Moran’s *Ι* value of ESV changes was 0.4724, and the spatial distribution of ESV changes had the strongest clustering characteristics. From 2010 to 2018, the Moran’s *Ι* value of ESV changes was only 0.2394, and the spatial distribution of ESV changes had the weakest clustering characteristics.

(2). Hot Spot Analysis

**Figure 6:**
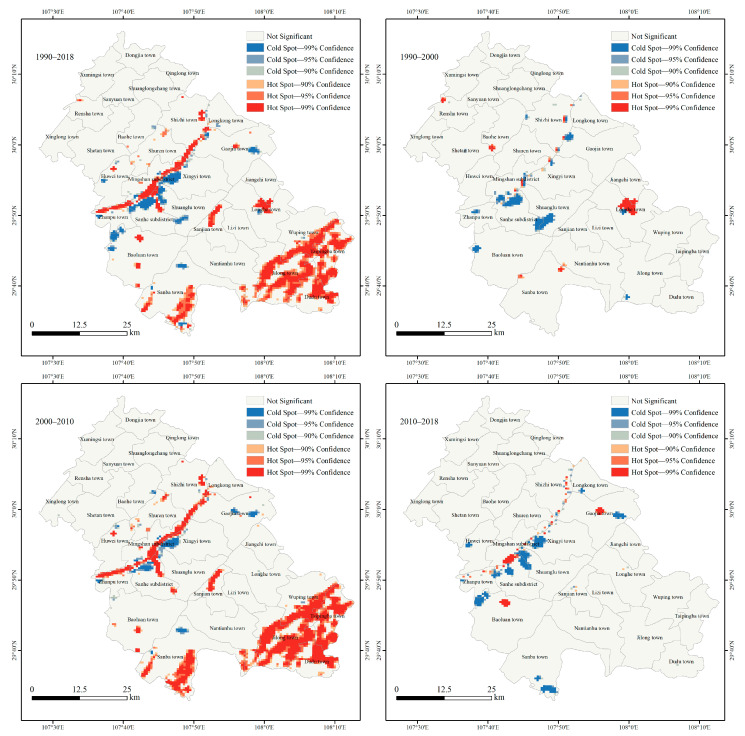
Hot spot spatial distribution pattern of ESV change in Fengdu County from 1990 to 2018.

Figure 6 shows the hot spot spatial distribution pattern of ESV changes in Fengdu County. From 1990 to 2018, most of the cold spots and hot spots of ESV changes in Fengdu County were distributed along the Yangtze River and to the south of the Yangtze River, which was consistent with the spatial distribution of ESV changes.

From 1990 to 2000, cold spots and hot spots of ESV changes were few and scattered. Among them, the cold spots were mainly distributed in the Mingshan subdistrict, Sanhe subdistrict, Zhanpu town, Baoluan town, Shuanglu town, and Gaojia town. This was mainly due to the expansion of US and ALS. The hot spots were mainly distributed in Longhe town. The reason is that the construction of Shiban Reservoir (since 1997) led to the rapid expansion of local WES; from 2000 to 2010, the cold spots and hot spots in Fengdu County increased significantly. As the TGD began to store water, hot spots were concentrated along the Yangtze River. At the same time, the increase in the water area of the Long River also led to the emergence of hot spots within the Sanhe subdistrict and Sanjian township. Due to the transition of grassland to forest with higher ESV per unit area, large areas of hot spots appeared in Sanba township, Jilong town, Wuping town, Taipingba township and Dudu township. In addition, due to the construction of the Danzitai reservoir (since 2003), hot spots also appeared in Baoluan town. The cold spots were mainly distributed in the Sanhe subdistrict, Baohe township, Xingyi town, Gaojia town and Sanba township. From 2010 to 2018, the cold spots and hot spots decreased compared with the previous period. The further expansion of the water area of the Yangtze River triggered the agglomeration of hot spots. The hot spots were mainly distributed in the Mingshan subdistrict, Baoluan town, and Gaojia town. The main reasons were the further expansion of the water area of the Yangtze River, and Jiangjiagou Reservoir (since 2015), Guantiangou Reservoir (since 2017), and Liziping Reservoir (since 2017), which have been built one after another. The cold spots were mainly distributed in Huwei town, Baoluan town, Sanhe subdistrict, Shuanglu town, Xingyi town, Gaojia town and Sanba township. The main reasons are the construction of the Fengdu Railway Station (since 2013) and the Fuling–Fengdu–Shizhu Expressway (since 2013), and the continuous growth of urban space and agricultural living space in each township. Overall, the cold spots and hot spots were the most widely distributed from 2000 to 2010, and this was the period with the most dramatic changes in ESV in Fengdu County.

## 4. Discussion

The reform of China’s ecological civilization system is the cornerstone of the reform of national land spatial planning. In the context of increasing emphasis on the development of an ecological civilization, ecosystem services have important reference significance for national land spatial planning. However, there is an objective contradiction between the complexity of the theory and methods of ecosystem services and the feasibility of national land spatial planning practices. How to integrate ecosystem services into the new spatial planning system has become an important task of China’s NLS governance [66,67]. This paper starts with ESV, an important measure of ecosystem services, and attempts to analyze the spatiotemporal change characteristics of ESV under the influence of NLSP change in Fengdu County. The conclusions can provide a scientific basis for promoting the multifaceted supporting role of ecosystem services in the formulation of national land spatial plans.

### 4.1. Effects of NLSP Change on ESV

The ESV of Fengdu County increased by CNY 11.10 × 10^8^ from 1990 to 2018. The construction and operation of the Three Gorges Reservoir and some small reservoirs has greatly increased the water ecological space in Fengdu County, which has made the most contribution to the total growth of ESV. This was similar to the research results in the upstream Xiong’an New Area [41]. In the ES, the large increase in forest has had a huge contribution to the total growth of ESV. Particularly, since 2000, with the implementation of the Grain for Green Project and Natural Forest Protection Program, the forest coverage rate has greatly increased, resulting in a significant increase in ESV. This was similar to the research results in northern Shaanxi [68]. The ESV per unit area of the construction space was negative. Over the past 28 years, a large number of other spaces have been transformed into construction space, which has had a large negative impact on the ESV of Fengdu County. This also reflects the problems existing in the development and utilization of NLSs in Fengdu County. Firstly, the development and utilization model of US pays too much attention to scale and speed and neglects the intensive use of space. Secondly, with the massive outflow of the rural population, the ALS has not decreased but has increased. Relevant studies have shown that this pattern of dysfunctional development of rural human–land relationships is widespread in China, and it is one of the main problems in China’s space governance [69,70,71].

In general, the spatiotemporal changes of ESV are processes that positively respond to the increase in ecological space but negatively respond to the expansion of urban space. With the completion and operation of the Three Gorges Reservoir and the long-term implementation of the Grain for Green Project, the water area and forest area of Fengdu County have basically stabilized. In the future, it will be difficult to significantly increase the ESV by increasing the water and forest. However, the urbanization process of Fengdu County will continue to advance, and it is foreseeable that ESV will face downward pressure in the future. Therefore, the NLSP should be guided to develop in the direction of ESV appreciation while ensuring that ESV does not depreciate.

We also found that ESV change shows obvious positive spatial autocorrelation characteristics, which indicates that NLSP change may have a certain spatial spillover effect on ESV. Moreover, NLSP changes may cause ESV gains and losses in the region. At the same time, they may also cause ESV increases and decreases in surrounding areas. The study of Lu et al. [72] reached a similar conclusion. The reason may be that NLSP changes have affected the material, energy, and information interactions between organisms and environmental components in the local ecosystem. The theory of landscape ecology shows that when the ES changes to AS and US, in which human activities are more intense, it will increase resistance to the migration and flow of species and energy between heterogeneous landscapes, which is not conducive to the progress of regional ecological processes [73], in turn leading to the weakening of ESV in surrounding areas. Therefore, preventing the expansion and penetration of spaces with higher ESV per unit area to spaces with lower ESV per unit area is an effective way to maintain the continuity of the ecosystem pattern and increase regional ESV.

### 4.2. Discussion on the Impact of the Three Gorges Dam on the Ecological Environment

This study mainly analyzed the impact of the construction of the TGD on ESV from the perspective of NLSP changes. From the research results, the construction of the TGD has increased the scale of water ecological space and has had positive significance for the regional ecosystem. However, it is worth discussing that the construction and operation of large dams also has a huge adverse impact on the ecological environment [74], which is confirmed by relevant studies in the Amazon Basin [75], Tennessee Valley [76] and the Mekong River Basin [77]. The TGD is the world’s largest hydro project, and is of great significance in flood control, power generation, and shipping. However, the TGD has also caused ecosystem degradation, water pollution, biodiversity reduction, downstream river erosion, geology disasters, and many other ecological hazards [78,79]. The Chinese government has implemented a series of policy interventions to mitigate adverse eco-environmental impacts of the TGD. Initially, during the construction of the TGD (1993–2002), many ecological programs were planed and enforced. The Transforming Sloping Cropland to Terraced Land (since 1993), Grain for Green Program (since 1998), Natural Forest Protection Program (since 1998), and the Comprehensive Plan on Prevention and Control of Geological Hazards in the Three Gorges Reservoir Area (since 2001) are a few examples. Subsequently, since the start of operation of the TGD in 2003, projects such as the Water Pollution Prevention and Control Plan in the Three Gorges Reservoir Area and the Upstream (2001–2010), and the Outline of the Water Pollution Prevention and Water Pollution Prevention and Control Plan in the Key Basins (2011–2015) have gradually been implemented. More than ten ecological operation trials were carried out simultaneously to rehabilitate “four domestic fish species (herring, grass carp, silver carp, bighead carp)”. These ecological programs have played a significant role in mitigating the negative ecological impacts of the TGD. From 1996 to 2016, 2118.47 km^2^ of sloping cropland were returned to forest or grassland, 2196 km^2^ of soil under erosion were curbed, and the forest coverage rate increased from 22% to 49% in the Three Gorges Reservoir Area. At the same time, water quality in the tributaries in the reservoir area improved, with the proportion of eutrophication being reduced from 39.4% in 2011 to 29.8% in 2016. Annual average spawning stocks of four domestic fish species increased by 137.4% in 2011–2016 on the basis of 2003–2010 levels [80]. In summary, we suggest that the pros and cons of the dams should be fully traded-off before construction. For dams that have already been built, it is necessary to carry out systematic ecological restoration measures, especially in some developing countries with increasing demand for water and energy but not enough awareness of ecological protection [81].

### 4.3. Policy Implications

Combined with our research, the following policy recommendations are suggested.

(1) This study concludes that NLSP is an important influencing factor of ESV, and ESV is an important basis for promoting NLSP to conform to the concept of ecological civilization. It is necessary to integrate ESV into the decision-making of national land space planning. Therefore, we suggest: (a) providing special training on ESV for spatial planners so that they can firmly grasp the relevant theories and evaluation methods of ESV; (b) based on relevant academic research, ESV should be included as a quantitative indicator in the work of delineating the “three zones and three lines” (three zones—ecological zone, agricultural zone, and urban zone; three lines—permanent basic farmland red line, urban development boundary, and ecological red line) and identifying key areas for ecological restoration [82]; and (c) monitoring and assessing the impact of the implementation of the national land spatial plan on the ESV to provide a basis for the revision of plans.

(2) Promoting the coordinated development of urban, agricultural, and ecological spaces is an important way to simultaneously achieve stable economic and social development and sustainable improvement of ecosystem services. Thus, we suggest the following: (a) It is necessary to strengthen the intensive use of urban space, fully tap the potential of existing urban land use, increase spatial compactness, and strictly enforce the control of urban development boundaries; (b) The protection of basic farmland should be strengthened, and sloping farmland and abandoned farmland should be gradually returned to forests. At the same time, rural residential land consolidation should be combined with the flow of urban and rural construction land indicators to maintain a balance between urban and rural construction land; (c) Land use control measures must be strictly implemented to protect the important ecological space from decreasing. At the same time, the integrated land consolidation and ecological restoration project of mountain–river–forest–field–lake–grassland should be implemented to improve the quality of the ecological environment.

(3) The spatial spillover effects of NLSP change on ESV should be fully considered. We suggest that a population withdrawal policy should be implemented in the ecological space, and the regional population should be encouraged to congregate in the urban spaces. At the same time, the development and construction activities in high ESV areas should be minimized. In addition, the urban space and agricultural living space should focus on the development of eco-friendly space uses on their natural edges, such as country parks and Linpan [83] (a kind of natural settlement of forests, water, houses, and fields widely distributed in southwestern China), so that they can be integrated with the surrounding ecosystem. Especially along the Yangtze River, it is even more necessary to plant a certain scale of ecological forests or build green parks to create ecological coastal zones to avoid pollution of the water environment of the Yangtze River from urban construction.

(4) The Three Gorges Reservoir area undertakes the important task of ecological environmental protection and restoration and has lost some opportunities for economic and social development to improve the service functions of the ecosystem. This has widened the gap in regional development and caused an imbalance between fairness and efficiency. For the sake of achieving regional fairness and sustainable development, we suggest taking ESV as the foundation for defining the regional ecological compensation relationship, determining the ecological compensation standard, and dividing the ecological compensation zones. At the same time, we have explored the establishment of a market-oriented and diversified ecological compensation mechanism for different regions and different principal parts to improve the enthusiasm and sustainability of ecological environmental protection.

### 4.4. Limitations

This study also has limitations. The ESV assessment method adopted in this study does not consider the impact of different use methods and use conditions of built-up areas on the ESV coefficient [84]. The negative impact of built-up spaces on ecosystem service functions mainly comes from human disturbance. The population density on urban land is relatively high, and the interaction between humans and land is strong. However, rural residential land carries a smaller population per unit area and causes less damage to the natural ecosystem of the land. In addition, idle rural residential land is subject to little human disturbance, and its negative impact on ecosystem service functions is almost negligible. Therefore, if the types of built-up land are subdivided and assigned value coefficients consistent with their ecological functions, the evaluation result of ESV will be more accurate. For other land use types such as forest, grassland, and water, the internal differences between them has less influence on the ESV coefficient. This is because these land use types belong to natural ecosystems, which are weakly disturbed by human activities, and the interaction between man and land is simple; the ESV coefficient mainly depends on the regional natural endowment [59]. At the county scale, regional natural endowments have a certain degree of homogeneity [85], and the difference in the spatial distribution of the internal structure and external form of the ecosystem is not significant.

The scope of application of the research results in this paper also has certain limitations. A significant negative relationship existed between topographic gradients and human disturbance. With the increase in altitude, the disturbance of human activities to the land ecosystem continued to weaken, and the value of ecosystem services showed an upward trend [86]. The terrain of Fengdu County is dominated by mountains, and the population is mainly distributed in mountain troughs with a flat terrain. In addition, in recent years, a large number of people have moved out of mountainous areas, which has further strengthened the differences in population distribution on topographical gradients [87]. Therefore, the spatial distribution of ESV in Fengdu County showed obvious imbalance. However, the topographical gradients of plain areas have little influence on human activities, and the difference in the spatial distribution of ESV may not be obvious. Therefore, the results of this research are not necessarily applicable to plain areas, such as the Amazon Plain, the North American Prairie, the Gangetic Plain, and the Northeast China Plain. However, the analysis conclusions of the ESV change mechanisms in this research are universal and can provide references for the management of ecosystem services in plain areas.

## 5. Conclusions

(1) From 1990 to 2018, the changes of NLSP in Fengdu County generally manifested in the transformation of AS into US and ES. US, ALS, VES, and WES increased, while APS and OES decreased. The newly added US and WES were mainly located along the Yangtze River, and the newly added ALS and VES were scattered.

(2) The spatiotemporal changes of ESV are processes that positively respond to the increase in ES but negatively respond to the expansion of US. From 1990 to 2018, the total ESV of Fengdu County showed a trend of continuous growth, with a total increase of CNY 11.10 × 10^8^, and the change rate was 9.33%. The significant increase in the ESV of the WES was the main reason for the increase in the total ESV of Fengdu County. The ESV gain area was mainly located along the Yangtze River and south of Fengdu County, and it has benefited from the implementation of ecological protection policies and the construction of the Three Gorges Reservoir and some small reservoirs. The ESV loss area was mainly located in Sanhe subdistrict, Shuanglu town, and Xingyi town on the south bank of the Yangtze River. The reason is that the construction of the new city invaded many other spaces.

(3) ESV and its change have a significant positive spatial autocorrelation. From 1990 to 2018, Moran’s *Ι* of ESV and its change in Fengdu County were all greater than 0, indicating that the spatial distribution of ESV and its changes showed strong spatial clustering characteristics. The spatial distribution of cold spots and hot spots of ESV changes at different stages was consistent with ESV changes, and these cold and hot spots were mainly located along the Yangtze River and to the south of the Yangtze River.

## Figures and Tables

**Table 1:** The classification of NLS in Fengdu County.

Primary Categories	Secondary Categories	Corresponding Land Use Type (Second-Level Categories)
Urban space (US)	—	Built-up (urban land, industrial and mining land)
Agricultural space (AS)	Agricultural production space (APS)	Cropland (paddy field, dry land)
	Agricultural living space (ALS)	Built-up (rural residential land)
Ecological space (ES)	Vegetation ecological space (VES)	Forest (closed forest land, open forest land, and other forest land) and Grassland (high coverage grassland, medium coverage grass, and low coverage grass)
	Water ecological space (WES)	Water (river, lake, reservoir, pond, and beach)
	Other ecological space (OES)	Unused land (bare land)

**Table 2:** Ecosystem service value per hectare of different land use categories in the study area. (Unit: CNY/hm^2^/year).

Land Use	Paddy Field	Dry Land	Forest	Grassland	Water	Built-Up	Unused Land
Ecosystem	Paddy field	Dry land	Broad-leaved	Shrub grass	Weter	—	Barren
Provisioning service	−3257.20	3094.90	3285.32	3174.83	314,672.47	−406.34	0
Regulating service	12,310.37	3521.59	39,376.18	32,601.97	7200.90	−10,653.50	377.79
Supporting service	988.79	2339.02	10,797.07	9769.31	4592.60	4138.55	82.22
Cultural service	218.70	145.80	2575.75	2332.75	352,787.22	9.47	24.30
Total	10,260.66	9101.31	56,034.32	47,878.86	679,253.19	−6911.82	484.31

**Table 3:** Changes in NLS structure in Fengdu County from 1990 to 2018.

Type		US	AS			ES			
			APS	ALS	Total	VES	WES	OES	Total
1990	Area/km^2^	1.95	1379.68	3.26	1382.94	1474.90	39.40	0.36	1514.66
	Proportion/%	0.07	47.58	0.11	47.69	50.87	1.36	0.01	52.24
2000	Area/km^2^	5.76	1373.02	5.03	1378.05	1474.10	41.27	0.35	1515.72
	Proportion/%	0.20	47.35	0.17	47.52	50.84	1.42	0.01	52.27
2010	Area/km^2^	13.71	1327.67	7.02	1334.69	1498.70	50.89	0.05	1549.64
	Proportion/%	0.47	45.81	0.24	46.05	51.71	1.76	1.83 × 10^−3^	53.47
2018	Area/km^2^	24.68	1317.37	8.24	1325.61	1495.37	52.33	0.05	1547.75
	Proportion/%	0.85	45.46	0.28	45.74	51.60	1.81	1.74 × 10^−3^	53.41
Rate of change/%		1165.64	−4.52	152.76	−4.15	1.39	32.82	−86.11	2.18
Annual rate of change/%		41.63	−0.16	5.46	−0.15	0.05	1.17	−3.08	0.08

**Table 4:** NLS transition matrix of Fengdu County from 1990 to 2018. (Unit: km^2^).

1990	2018					
	US	APS	ALS	VES	WES	OES
US	0.76	0.02	0	0.01	1.17	0
APS	18.24	1246.66	5.39	100.45	8.35	0
ALS	0.21	0.49	2.45	0.10	0	0
VES	5.24	68.44	0.33	1393.57	5.04	0.01
WES	0.23	1.18	0.06	0.36	37.56	0
OES	0	0.12	0	0.01	0.19	0.04

**Table 5:** ESV of Fengdu County from 1990 to 2018 (unit: 10^8^ CNY).

Ecosystem Service	1990	2000	2010	2018
Provisioning service	19.13	19.70	22.67	23.09
Regulating service	63.57	63.41	65.85	65.54
Supporting service	18.43	18.43	18.92	18.92
Cultural service	17.83	18.48	21.99	22.49
Total	118.95	120.03	129.43	130.05

**Table 6:** The ESV of different NLS types in 1990–2018 (unit: 10^8^ CNY).

Type	US	AS			ES			
		APS	ALS	Total	VES	WES	OES	Total
1990	−0.01	12.98	−0.02	12.96	79.24	26.77	1.72 × 10^−4^	102.61
2000	−0.04	12.92	−0.03	12.89	79.15	28.03	1.72 × 10^−4^	103.61
2010	−0.09	12.51	−0.05	12.46	82.50	34.57	2.44 × 10^−5^	111.86
2018	−0.17	12.42	−0.06	12.36	82.31	35.55	2.44 × 10^−5^	112.82
change	−0.16	−0.57	−0.03	−0.60	3.07	8.78	−1.48 × 10^−4^	10.21

**Table 7:** Moran’s *I* value of ESV in Fengdu County.

Index	1990	2000	2010	2018	1990–2000	2000–2010	2010–2018	1990–2018
Moran’s *Ι*	0.6324	0.6214	0.6398	0.6437	0.34674	0.4724	0.2394	0.4542
*p*-value	<0.001	<0.001	<0.001	<0.001	<0.001	<0.001	<0.001	<0.001
z-scores	132.5123	134.4064	128.2431	147.6582	115.3241	123.1716	89.9226	96.9496

## Data Availability

Not applicable.

## Abbreviations

The abbreviations in this article:

ESV	Ecosystem Service Value
NLS	National Land Space
NLSP	National Land Space Pattern
TGRA	Three Gorges Reservoir Area
TGD	Three Gorges Dam
US	Urban space
AS	Agricultural space
ES	Ecological space
APS	Agricultural production space
ALS	Agricultural living space
VES	Vegetation ecological space
WES	Water ecological space
OES	Other ecological space
NPP	Net Primary Productivity

## Appendix A

The “Two Mountains” Theory is a scientific conclusion put forward in 2005 by Jinping Xi, the President of China. Its core idea is that “lucid waters and lush mountains are invaluable assets”, which means that a good ecological environment is the most inclusive factor promoting human well-being, and maintaining the ecological environment means maintaining productivity.
